# Editorial: Tissue Repair and Regenerative Mechanisms by Stem/Progenitor Cells and Their Secretome

**DOI:** 10.3389/fmed.2019.00011

**Published:** 2019-01-31

**Authors:** Roberto Gramignoli, Fabio Sallustio, Darius Widera, Nathanael Raschzok

**Affiliations:** ^1^Department of Laboratory Medicine, Karolinska Institutet, Stockholm, Sweden; ^2^Department of Basic Medical Sciences, Neuroscience and Sense Organs, University of Bari Aldo Moro, Bari, Italy; ^3^Stem Cell Biology and Regenerative Medicine Group, Reading School of Pharmacy, University of Reading, Reading, United Kingdom; ^4^Department of Surgery, Charité - Universitätsmedizin Berlin, Berlin, Germany

**Keywords:** tissue repair and regeneration, regenerative mechanism, stem cells, progenitor cells, secretome

Regenerative medicine is a branch of translational research which is energizing and empowering clinical practice. A multitude of novel approaches proposed during the recent years is slowly but dramatically transforming the health care system, harnessing the power of repairing, replacing, restoring, and regenerating human organs and tissues affected by various degenerative disorders and diseases.

During the twentieth century, hundreds of thousands of patients with end-stage diseases have been rescued by solid organ transplants as ultimate treatment option. In the past 3 decades, cell-based therapies have been gaining importance since they can contribute to regeneration of failing organs or damaged tissues by direct replacement of the lost cells or by facilitating the body's natural regenerative processes by removing roadblocks. A growing armamentarium of therapeutic options, spanning from bioartificial organs and tissues, stem and progenitor cells, biomaterials, cell secretome, and extracellular vesicles have become available as medical treatments substituting the standard pharmaceutics.

Examples of “classical” cellular therapies are peripheral blood stem cell or stromal cell transplantations, and more recently, allogenic hepatocyte or pancreatic islet transplants.

While pancreas transplantation remains the gold standard in diabetes patients where the insulin injection fails to control symptoms, transplantation of islets of Langerhans has been recognized as a successful cell-based treatment in type 1 diabetes. The mechano-enzymatic separation of endocrine tissue from the exocrine pancreatic parenchyma required several decades to become a standardized clinical approach approved by Medical Product Agencies worldwide. A systematic review, describing all the significant obstacles and solutions currently proposed to overcome and optimize pancreatic islet transplantation has been compiled by Bottino et al. The Pittsburgh-based group with over two decades of clinical experience addressed these “roadblocks” including, but not limited to, availability of clinical grade reagents, reduced amount and quality of pancreas as cell source, quality control evaluation, and hypothermic storage of islets prior to their transplantation as well as immunosuppression protocols commonly used in the recipients.

Another interesting cell-based approach, which has seen its clinical use waxed and waned throughout past 25 years, is hepatocyte transplantation. Hepatocyte transplantation has gained importance as alternative or supportive treatment to orthotopic liver transplantation for congenital or fulminant liver disease. However, despite initial promising clinical results, long-term survival of the transplanted cells has been identified as the major obstacle for broader application of this cellular approach. Intraperitoneal transplantation of human liver cells, encapsulated in alginate microbeads has been proposed as a promising clinical option by Iansante et al. The authors, based at the King's College Hospital group in London combined the protective effects of alginate beds with the supportive function of mesenchymal stromal cells (MSC). This combined approach appears to exhibit protective effects on primary human hepatocytes, not only in terms of survival but also regarding the central physiological functions of this cell type namely metabolic control and detoxification.

All the metabolic functions performed by hepatocytes, fundamental for body homeostasis as well as their unique capacity to regenerate, have recently been linked to secondary messengers, including calcium. Calcium is a versatile secondary messenger involved in several hepatic functions, whose dysregulation signaling is commonly associated with both acute and chronic diseases. Consequently, intracellular calcium control has been emerging as a therapeutic target for regenerative and curative therapies, as eloquently described by Oliva-Vilarnau et al.

As introduced above, MSCs have found clinical application in several pathological conditions. Notably, their therapeutic potential is nowadays believed to be a consequence of a combination of integration and paracrine modulation of endogenous regeneration mediated by a multitude of secreted immunomodulatory, anti-inflammatory, and regenerative factors (cellular products). A case study by Salikhov et al. highlights the regenerative potential of adipose tissue-derived MSCs in a 36-year-old patient suffering from osteochondral femoral lesions. Eight months post-injury, autologous adipose-derived stromal cells have been injected in combination with fibrin sealant fixative and the clinical picture has been followed up for 24-months. This new method combining a biomaterial (fibrin) as a cell-fixative and MSCs could represent a potential treatment option for osteochondral joint lesions.

The translation of regenerative medicine therapies from bench to bedside has been a long and windy road, paved by lack of convincing data on the safety and uncertain levels of efficacy in recognized biological/medical models and systems. Currently, there is a plethora of unproven or insufficiently proven cell-based treatments commercially available for a variety of conditions. While there is a long history of advances in biomedical research, there is an equally long history of research fraud, lack of dignity, honesty, compassion, and respect for patients, contributing to the commercial exploitation of unproven and often harmful (bio-)medical interventions.

Relevant pre-clinical models of disease are of fundamental importance for acquiring convincing safety and efficacy data before translating such therapies into the human system (as evidenced by recent medical scandals in regenerative medicine area).

Data generated in irrelevant models are also a concern, as they could give a false sense of risk or safety that may heavily impact on the appropriate use of a cell-based product. Due to the human origin of most cell-based medical products, the existing preclinical small animal models are frequently not an ideal tool for prediction of the effects in patients, and need to be adapted on a case-case basis. Species differences between small animals (rodents) need to be considered, while large animals are difficult to handle and generate substantial costs, making their use less common in proof-of-concept studies.

A variety of preclinical studies have highlighted the protective role performed by transplanted MSC. In this context, a review by Torres Crigna et al. summarizes our current knowledge on the mechanistic aspects of stromal cell-mediated renoprotection. Here, the authors conclude that stromal cells exhibit protective and pro-regenerative effects as renal precursors and via paracrine factors including trophic, anti-inflammatory, and immunomodulatory molecules.

The field of cell transplantation has evolved into a sophisticated profession requiring knowledge and skills not only in traditional clinical and laboratory medicine, but also in systems and molecular biology, materials technology and bioengineering, veterinary medicine, epidemiology, quality management and automation, statistics, risk analysis, and bioethics. Currently, cellular therapies are largely identified as rather sophisticated procedures under strict environmental control that can only be ensured in certified laboratory facilities. Within such cell factories, well-defined cell (sub)populations undergo some degree of manipulation or engineering, and clear documentation of prospectively defined, measurable clinical outcomes would establish safety and efficacy. In addition to the substantial health risks, patients and their families are often exposed to financial risks, dashed hopes and other forms of psychological harm. There is genuine concern that the growing market size of these unproven and possibly harmful cell-based interventions may negatively affect the legitimate development of evidence-based cellular therapies.

Cellular medical approaches are revolutionizing the traditional biotechnology and pharmaceutical industries with the promise of ground-breaking new therapies for devastating and costly conditions. Novel cellular therapies are often expensive and offered outside the umbrella of established clinical care. Recently, the U.S. Food and Drug Administration approved first cellular immunotherapies and gene therapies, alongside respective reimbursement by the U.S. Centers for Medicare & Medicaid Services. This has resulted in record-breaking investments and acquisitions in the sector. The income for cell-based therapies was predicted to become the one dominating the global regenerative medicine market in 2016, and currently it has been estimated to be a multi-billion business, with a market realistically expected to reach 39 billions of American dollars by 2021 (1). Currently, there are more than 800 companies worldwide focused on Gene and Cell Therapies, and Tissue Engineering (2). Up to date, over 950 clinical trials applying cell, gene, and tissue are ongoing worldwide (2). Although the dynamic field of regenerative medicine offers great hope for future treatment of previously incurable conditions, the scientific community must ensure that all cell-based therapy suffice the criteria of no toxicity and tumourigenicity and have an efficacy higher than placebo in relevant animal models before entering the market or even being applied as compassionate use in patients. Moreover, a sufficient level of information on the mode of action should be provided. This could not only lead to a development of robust, cell-based therapeutics but also prevent unproven stem cell therapies that could harm the patients and the credibility of the field. Thus, we as Guest Editors urge that regenerative medicine should be evidence- and not enthusiasm-based.

## Author Contributions

All authors listed have made a substantial, direct and intellectual contribution to the work, and approved it for publication.

### Conflict of Interest Statement

The authors declare that the research was conducted in the absence of any commercial or financial relationships that could be construed as a potential conflict of interest.

## References

[B1] Regenerative Medicines Market by Therapy Product & Application 2021. (2017). Available online at: https://www.marketsandmarkets.com/Market-Reports/regenerative-medicine-market-65442579.html

[B2] Q1 2018 Data Report - Alliance for Regenerative Medicine. (2018). Available online at: https://alliancerm.org/publication/q1-2018/

